# Editorial: Regulation of Immunity by Non-Immune Cells

**DOI:** 10.3389/fimmu.2021.770847

**Published:** 2021-09-21

**Authors:** Teruki Dainichi, Kenji Kabashima, Ivaylo I. Ivanov, Yoshiyuki Goto

**Affiliations:** ^1^Department of Dermatology, Kagawa University Faculty of Medicine, Kagawa, Japan; ^2^Department of Dermatology, Kyoto University Graduate School of Medicine, Kyoto, Japan; ^3^Singapore Immunology Network (SIgN) and Skin Research Institute of Singapore (SRIS), Agency for Science, Technology and Research (A*STAR), Biopolis, Singapore; ^4^Department of Microbiology and Immunology, Vagelos College of Physicians and Surgeons, Columbia University, New York, NY, United States; ^5^Division of Molecular Immunology, Medical Mycology Research Center, Chiba University, Chiba, Japan; ^6^Division of Mucosal Symbiosis, International Research and Development Center for Mucosal Vaccines, The Institute of Medical Science, The University of Tokyo, Tokyo, Japan

**Keywords:** nonimmune cell, mucosal tissue, skin, fat, stroma, joints, blood vessels

## Introduction

The immune system is a highly sophisticated system that governs a major part of the protective and regenerative responses of the host against external and internal microbes, poisons, and other dangers. Classical innate and acquired immune cells include dendritic cells, macrophages, neutrophils, eosinophils, innate lymphoid cells, T cells, and B cells. B and T cells are activated by specific antigens; they organize the most effective type of immune response by eliminating harmful events. Phagocytic cells, such as dendritic cells and macrophages, play essential roles in both (i) the antigen-specific activation of lymphocytes and (ii) the situation-specific functional deviations of lymphocytes. In contrast, non-immune cells, which include epithelial cells, epidermal keratinocytes, mesenchymal cells, stromal cells, synoviocytes, and neurons, are expected to participate in the host’s defense system not just as structural architectures, but also as regulators and effectors of its protective immune response. For example, the molecules released from damaged non-immune cells can affect several types of immune responses. Furthermore, the *de novo* production of bioactive mediators by non-immune cells, in response to several stimuli, can be involved in these processes. Therefore, defects in the abilities of non-immune cells to mediate immune regulation may be involved in the pathogenicities of a series of inflammatory diseases. During the process of immune regulation, non-immune cells must sense each type of danger signal appropriately, interpret it precisely, and activate the most appropriate type of immune response to overcome said danger. However, the precise cellular mechanisms involved in this process have not yet been fully elucidated. Thus, there is a need for a review series that explores the consideration of a common mechanism of immunity regulation by non-immune cells.

Here, we provide a cutting-edge collection of Review and Mini Review articles that discuss the roles and mechanisms of immune modulation by non-immune cells, beyond organs, tissues, and cell types. Our goal is to stimulate discussion regarding the common and essential roles of non-immune cells in orchestrating their host’s protective responses and inflammatory processes, and to further our knowledge in this field.

## Mucosal Tissues

The gastrointestinal tracts are constitutively exposed by countless numbers of antigens, including commensal and pathogenic microorganisms. The epithelial monolayer that covers the gastrointestinal tract contains luminal antigens in the lumen. Therefore, the disruption of the epithelial layer predisposes patients to the development of inflammatory bowel disease (IBD). Internal nuclear factor-κB (NF-κB) signaling is a molecular signaling mechanism that regulates the homeostasis of intestinal epithelial cells (IECs). Garcia-Carbonell et al. summarized the molecular network of epithelial NF-κB signals downstream of the tumor necrosis factor (TNF) receptor. They also described the role that NF-κB signaling plays in IECs regarding the maintenance of the epithelial barrier system. They highlighted that the dysregulation of NF-κB signaling predisposes the development of IBD.

Goto discussed that IECs have the potential to modulate the mucosal immune system and construct a physical barrier. They also described the immunomodulatory effects of IECs in response to luminal antigens, especially to commensal and pathogenic microorganisms. At the same time, IECs produce antimicrobial molecules, including bactericidal peptides, immunoglobulin (IgA), and carbohydrate moieties. These molecules are induced by cytokines and molecules that are produced by immune cells in the lamina propria. Therefore, IECs work as bidirectional transducers of signals from luminal antigens and gut immune cells to maintain gut homeostasis.

Xue et al. highlighted that in the intestine, non-immune cells, such as IECs and enteric neurons, produce neurotransmitters such as dopamine. Neurotransmitters have been reported to have various immunomodulatory functions that are thought to be associated with the development and regulation of immunological diseases. They also provided evidence that intestinal dopamine, induced by commensal microbes, suppresses liver injury through the D1-like receptor-protein kinase A (PKA) signals in invariant natural killer T (iNKT) cells (Xue et al.). This suggests that the interplay between gut microbes and the host’s nervous and immune systems plays an important role in the prevention of autoimmune hepatitis.

Kurashima et al. discussed that, in the intestine, mesenchymal stromal cells, IECs, and immune cells create a network system. Although the interplay between IECs and immune cells has been extensively investigated, our understanding of the characteristics and functions of intestinal stromal cells is limited. They took a broad view in summarizing evidence linking the roles of stromal cells, which modulate the function and differentiation of IECs, and mucosal immune cells (Kurashima et al.). One mechanism that highlights the role of stromal cells in the modulation of the immune system is that stromal cells activated by lymphotoxins produce cytokines and chemokines, which in turn recruit lymphocytes. This subsequently induces the organogenesis of secondary lymphoid tissues, such as Peyer’s patches and mesenteric lymph nodes.

Hirahara et al. highlighted that the lungs also contain representative mucosal tissue composed of epithelial cells, stromal cells, and immune cells. They provided an elegant illustration of the molecular mechanism underlying the induction of inducible bronchus-associated lymphoid tissue (iBALT). In the context of allergic inflammation, interleukin (IL)-33 activates ST2+ memory CD4+ T cells to induce IL-5 and amphiregulin. These cytokines accelerate fibrosis and the pathology of asthma. Therefore, the interactions between immune and epithelial/mesenchymal cells are critical for the development of chronic lung inflammation.

## Skin

Zhang revealed that epidermal keratinocytes are located at the outermost position in the skin and are the first responders to external agents and skin injuries. They focused on the potential roles of type 1 interferons (IFNs) in psoriasis, which is one of the most common chronic inflammatory diseases. Skin injuries can rapidly induce IFNβ from keratinocytes and IFNα from dermal plasmacytoid dendritic cells, through distinct mechanisms. IFNβ derived from keratinocytes promotes dendritic cell maturation and subsequent T-cell proliferation, triggering the development of psoriasis.

Dainichi et al. discussed that keratinocytes can be involved in the organization of immune responses of the skin through two phases: the initiation of primary immune responses and the propagation of secondary responses; this gives rise to the loop of chronic inflammation. TNF receptor-associated factor 6 (TRAF6) is a ubiquitin E3 ligase that is essential for various receptor signaling pathways that activate NF-κB and mitogen-activated protein kinase (MAPK). The above-mentioned mini review describes the roles of TRAF6 in epithelial tissues, including keratinocytes, in both epithelial primary and secondary responses.

## Fat and Stroma

West highlighted that stromal cells complement the functions of classical immune cells by sensing pathogens and tissue damage, coordinating leukocyte recruitment and function, and promoting immune response resolution and tissue repair. Several members of the IL-6 cytokine family mediate crosstalk between stromal and immune cells; they play diverse roles in numerous inflammatory and homeostatic processes. The above-mentioned review summarizes our current understanding of how IL-6 family cytokines control stromal-immune crosstalk in healthy and diseased hosts, and how these interactions can be leveraged for clinical benefit.

Wong et al. prepared a mini review discussing the subcutaneous tissue, which forms an uninterrupted layer throughout the body in humans. Obesity leads to the upregulation of pro-inflammatory adipokines, and to the downregulation of anti-inflammatory adipokines from adipocytes. This results in the activation of the stromal vascular fraction, such as macrophages, in adipose tissue. Said mini review sheds light on the crosstalk between adipose and immune cells in psoriasis.

## Joints

Yoshitomi discussed the synovial tissue: a membranous, non-immune, organ-lining joint cavity. Fibroblast-like synoviocytes (FLSs) are the dominant non-immune cells in synovial tissues (Yoshitomi). FLSs mainly contribute to joint destruction in chronic inflammatory diseases, such as rheumatoid arthritis (RA), *via* multiple mechanisms. Their mini review describes the new findings and mechanisms underlying the regulation of immune reactions by non-immune FLS, and highlights their roles in the development of chronic inflammation.

## Blood Vessels

Hu et al. highlighted that the mechanisms of atherogenesis are currently largely undefined. However, it has been demonstrated that disease progression involves crosstalk between immune cells and both endothelial cells and vascular smooth muscle cells (VSMCs). VSMCs maintain the integrity of the arterial wall in the media layer of arteries. They also participate in the remodeling of the arterial wall in atherosclerosis, throughout all stages of the disease. The said mini review focuses on the roles that VSMCs play in atherosclerosis immunity by organizing artery tertiary lymphoid organs (ATLOs).

## Concluding Remarks

We thank the contributors and dedicated reviewers for their efforts and generous enthusiasm. Although this Research Topic does not fully capture the full breadth of activity in the regulation of immunity by non-immune cells, we hope that the series of articles identified will stimulate the communication and integration of this concept, among both immunologists and non-immunologists. We believe that our article makes a significant contribution to the literature because of its timeliness, succinctness, and the need to highlight this important direction of study.

## Author Contributions

All authors contributed to the article and approved the submitted version.

## Conflict of Interest

The authors declare that the research was conducted in the absence of any commercial or financial relationships that could be construed as a potential conflict of interest.

## Publisher’s Note

All claims expressed in this article are solely those of the authors and do not necessarily represent those of their affiliated organizations, or those of the publisher, the editors and the reviewers. Any product that may be evaluated in this article, or claim that may be made by its manufacturer, is not guaranteed or endorsed by the publisher.

